# First Insight into the Genome Sequences of Two Linezolid-Resistant *Nocardia farcinica* Strains Isolated from Patients with Cystic Fibrosis

**DOI:** 10.1128/genomeA.01212-17

**Published:** 2017-11-16

**Authors:** Sylvia Valdezate, Sara Monzón, Noelia Garrido, Angel Zaballos, María J. Medina-Pascual, José M. Azcona-Gutiérrez, Begoña Vilar, Isabel Cuesta

**Affiliations:** a Department of Bacteriology, National Centre of Microbiology, Reference and Research Laboratory for Taxonomy, Instituto de Salud Carlos III, Majadahonda, Madrid, Spain; b Bionformatics Unit, Core Scientific and Technical Units, Instituto de Salud Carlos III, Majadahonda, Madrid, Spain; c Genomics Unit, Core Scientific and Technical Units, Instituto de Salud Carlos III, Majadahonda, Madrid, Spain; d Hospital Universitario San Pedro, Logroño, Spain; e Hospital Universitario de Cruces, Barakaldo, Spain

## Abstract

The draft genome sequences of two *Nocardia farcinica* strains isolated from two patients with cystic fibrosis (CF), resistant to trimethoprim/sulfamethoxazole and linezolid, are reported here. The estimated genome sizes were 5.8 Mb with a 70.63% G+C content. Transposases from *Tn*916 were detected, but not 23S rRNA mutation (G2576T) related to linezolid resistance.

## GENOME ANNOUNCEMENT

*Nocardia* spp. are environmental bacteria that cause pulmonary infections similar to tuberculosis, and even disseminated disease (1, 2). *Nocardia farcinica* causes invasive infections with cerebral involvement (55%) and high mortality (35%) (1, 3). In Spain, resistance to trimethoprim/sulfamethoxazole (the cornerstone of treatment) in *N. farcinica* runs at 45% (4). Linezolid, with excellent bioavailability, provides an efficient alternative (1, 5). From childhood, patients with cystic fibrosis (CF) suffer frequent lung infections. From these patients, two *N. farcinica* strains (drug pattern type V [6]) highly resistant to trimethoprim/sulfamethoxazole and linezolid (MICs ≥32:608 and ≥256 µg/mL, respectively), were sequenced for a better understanding of their genetic background.

Paired-end libraries (Nextera-XT DNA library preparation kit, Illumina 1.9), were adapted and sequenced at 2 × 150 using Illumina NextSeq500. After quality control analysis involving fastQC v0.11.3 (http://www.bioinformatics.babraham.ac.uk/projects/fastqc/) and Trimmomatic v0.36 software (7), 3.1 million to 3.6 million paired-end reads of 50 to 151 bp were retrieved. Read assembly was performed using SPAdes v3.8.0 software (8) (kmer = 77), and the quality was evaluated using QUAST software (9).

The draft genomes of the strains, named CNM20080921 and CNM20091955, were both 5.8 Mbp long and had a G+C content of 70.63% (178 and 113 contigs of ≥500 bp; maximum, 674,734 and 1,141,487 bp; *N*_50_ value, 167,048 and 453,514 bp). Analysis using ANI software (10) revealed them to show 99.98% sequence similarity, and 99.28% similarity to *N. farcinica* NCTC11134 (GenBank accession number, NZ_LN868938). The assembled sequences were annotated using Prokka 1.12-beta software (11), which predicted 6,502 and 6,455 genes (6,428 and 6,381 protein-coding genes and 427 and 419 signal peptides, respectively), and 6 rRNAs, 67 tRNAs, and 1 transfer-messenger RNA (tmRNA) for both strains.

The following potential resistance genes (https://ardb.cbcb.umd.edu/, http://nocardia.nih.go.jp) were detected in both strains: *bmr*, *stp*, *marR*, *mtdL*, *norM*, *emrB*, *emrY*, FAR-1, *ampC*, *aacA-aphD*, *aph(3′)*, *ant1*, *neo*, *fstH*, *htpX*, *murA*, *ermH*, *tetA*, *tetC*, *tetR*, *mtdH*, *dfrA26*, *vanW*, *bac*, *qacA*, *sugE*, *acr3*, *ohrA*, *drrA*, *drrB*, *drrC*, *carA*, *merR*, and *yfmO*; *rmlA* was detected in CNM20091955 only. Detected resistant genes by PCR sequencing of boiled extract of both strains (12), e.g., *int1-3*, *sul1-2*, *ermTR*, *ermB*, and *tetO* (and *mefO* for CNM20080921), were not detected by srst2 (13) and ARGANNOT (14) software (only *int1*, *clmA*, and FAR-1 were detected). Neither of the two 23S rRNA copies showed the G2576T mutation (*Escherichia coli* numbering) allowing linezolid resistance. However, one allele of CNM20080921 showed the change G2608A. Mobile element-related sequences, such as *IS*5376/*IS*6110, *tnp*R (resolvase) guaA (integration hot spot), three IS3-family transposases, and two transposases from *Tn*916, were identified in both strains.

Similar virulence contents were detected such as mammalian cell entry protein, ESX-1, and others (*fbpA*, *fbpB*, *fbpC*, *sodC*, *katA*, *katE*, *katG*, *ideR*, *nbtB*, *nbtD*, *nbtE*, *nbtF*, *nbtG*, *nbtS*, *aphC*, *aphD*, *narG*, *oxyR*, *ndk*, *ptpA*, *tlyA*, and *tlyC*) (15). The biosynthetic gene clusters for CNM20080921 and CNM20091955 were 7 and 9 nonribosomal peptide synthases, 4 and 5 polyketide synthases, 5 terpene cyclases, and ectoine and nocobactin genes, respectively (16). Clustered regularly interspaced short palindromic repeats (CRISPR) (12 and 11, respectively) were found in each one. In both strains, the same incomplete prophage (7.4 Kb, 71.39% G+C) was identified (17).

Given their exposure to viral/bacterial infections and frequent antimicrobial treatment, the pulmonary microbiomes of patients with CF are fragile. If lung transplantation is to be successful in such patients, the surveillance of *Nocardia* species is needed (see reference 18).

### Accession number(s).

This whole-genome shotgun project has been deposited at DDBJ/ENA/GenBank under the accession numbers NXEH00000000 and NXEG00000000 for CNM20080921 and CNM20091955, respectively. The versions described in this paper are versions NXEH01000000 and NXEG01000000.
